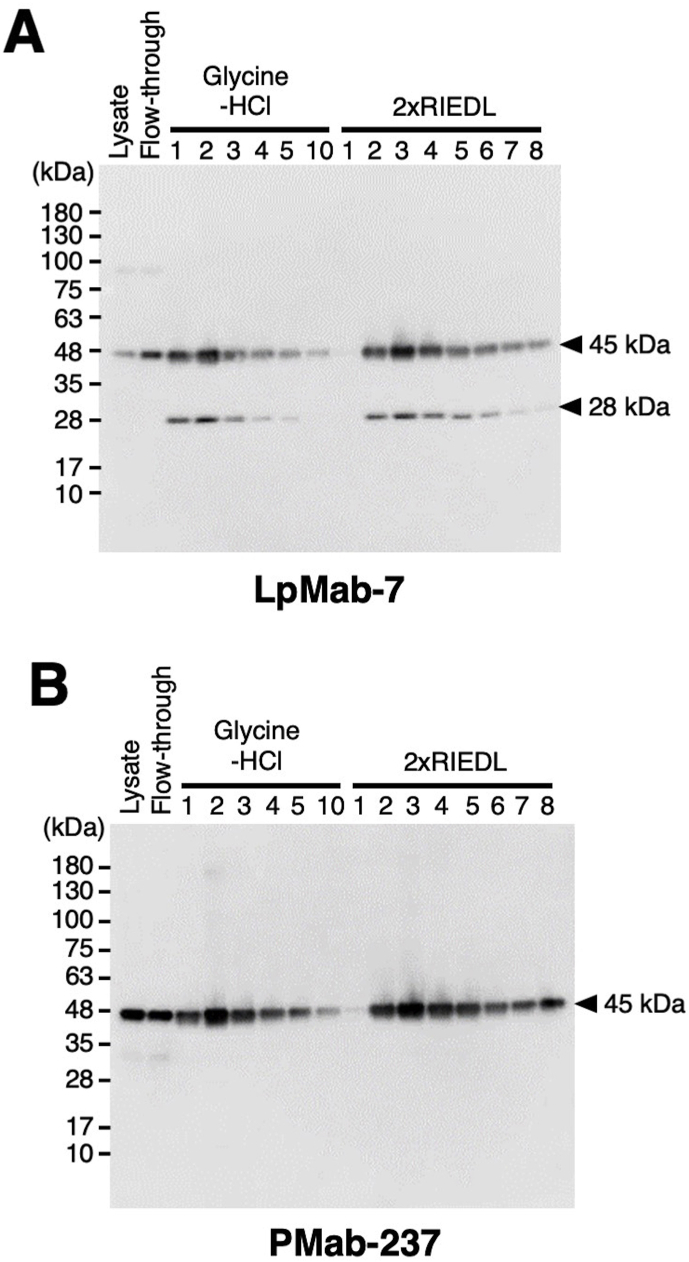# RIEDL tag: A novel pentapeptide tagging system for transmembrane protein purification

**DOI:** 10.1016/j.bbrep.2020.100780

**Published:** 2020-07-17

**Authors:** Teizo Asano, Mika K. Kaneko, Yukinari Kato

**Affiliations:** a Department of Antibody Drug Development, Tohoku University Graduate School of Medicine, 2-1 Seiryo-machi, Aoba-ku, Sendai, Miyagi, 980-8575, Japan; b New Industry Creation Hatchery Center, Tohoku University, 2-1, Seiryo-machi, Aoba-ku, Sendai, Miyagi, 980-8575, Japan

**Keywords:** Peptide tag, Protein purification, Monoclonal antibody, RIEDL tag, Podoplanin, CD20, mAb, monoclonal antibody, PBS, phosphate-buffered saline, wPDPN, whale podoplanin

## Abstract

Affinity tag systems are an essential tool in biochemistry, biophysics, and molecular biology. Although several different tag systems have been developed, the epitope tag system, composed of a polypeptide “tag” and an anti-tag antibody, is especially useful for protein purification. However, almost all tag sequences, such as the FLAG tag, are added to the N- or C-termini of target proteins, as tags inserted in loops tend to disrupt the functional structure of multi-pass transmembrane proteins. In this study, we developed a novel “RIEDL tag system,” which is composed of a peptide with only five amino acids (RIEDL) and an anti-RIEDL monoclonal antibody (mAb), LpMab-7. To investigate whether the RIEDL tag system is applicable for protein purification, we conducted the purification of two kinds of RIEDL-tagged proteins using affinity column chromatography: whale podoplanin (wPDPN) with an N-terminal RIEDL tag (RIEDL-wPDPN) and human CD20 with an internal RIEDL tag insertion (CD20-_169_RIEDL_170_). Using an LpMab-7-Sepharose column, RIEDL-wPDPN and CD20-_169_RIEDL_170_ were efficiently purified in one-step purification procedures, and were strongly detected by LpMab-7 using Western blot and flow cytometry. These results show that the RIEDL tag system can be useful for the detection and one-step purification of membrane proteins when inserted at either the N-terminus or inserted in an internal loop structure of multi-pass transmembrane proteins.

## Highlights

• We established a novel RIEDL tag system, composed of RIEDL peptide and LpMab-7 mAb.
• The RIEDL tag system is applicable for protein purification, as well as FCM and WB.
• The RIEDL tag, inserted into a loop structure of CD20, was detected by LpMab-7.
• RIEDL-tagged proteins were efficiently purified using 2 × RIEDL peptide.

## 1 Introduction

Protein purification and detection are essential techniques for protein research, including for the determination of structure and elucidation of function [1]. There exist several methods for protein purification, such as hydrophobic interaction chromatography, ion exchange chromatography, and affinity chromatography [2]. However, many steps are required to isolate a protein from tissues or cells, and it is often difficult to obtain highly purified proteins. To resolve these problems, several tagging systems have been developed [3,4]. By using an exogenous tag, a target protein can be purified directly from tissue or cell lysates, which can express recombinant tagged proteins in one or a few steps.

Affinity tag systems are classified into the “protein tag system” and the “epitope tag system.” Protein tags, including glutathione *S*-transferase (GST) [5], green fluorescent protein (GFP) [6], and maltose binding protein (MBP) [7], are useful for soluble protein expression; however, these tags can affect the functions of a target protein, as these tags have high molecular weights. Epitope tags, such as FLAG [8], HA [9], Myc [10], and PA [11], are composed of a short polypeptide, which acts as the tag, and are recognized using anti-tag antibodies that exhibit high specificity because of the antigen-antibody interaction. Moreover, epitope tags have low molecular weights, and are therefore not likely to affect the target protein's structure. A tag system should be selected by considering the advantages of each system. The FLAG tag is one of the useful epitope tag systems for protein purification and detection [8]. Although the FLAG tag can be added to the N- or C-terminus of a target protein, it is not applicable for immunoprecipitation assays when it is inserted into a loop structure [1]. When a Myc tag was inserted into arrestin, anti-Myc antibodies were shown to function as a competitor for arrestin binding to rhodopsin [12]. HA and T7 tags, when inserted into KdpD, could be recognized by antibodies in an immunoprecipitation assay [13].

In our previous studies, we established the PA tag [11] and MAP tag systems [14]. The PA tag was successfully inserted into a loop structure in integrin and Sema3A, and proved useful for flow cytometry and immunoprecipitation assays [1]. The MAP tag can also be inserted into the loop regions of various proteins, and tag antibody interactions were maintained [15]. Although several tag systems can be inserted into the loop regions of multi-pass transmembrane proteins and are applicable for some biological assays as described above, much smaller epitope tags are needed to minimize the effects of the inserted peptides on the proteins. In this study, we established a novel tag system, called the “RIEDL tag system,” composed of LpMab-7 (an anti-human podoplanin mAb) and a peptide, RIEDL, consisting of only five amino acids, and investigated the utility of this tag system in protein purification.

## 2 Materials and methods

### 2.1 Plasmids

Synthesized DNA (Eurofins Genomics KK, Tokyo, Japan) encoding wPDPN (accession No.: XM_007104824.2) plus an N-terminal RIEDL tag, which is recognized by an anti-RIEDL tag mAb (LpMab-7), was subcloned into a pCAG-Neo vector (FUJIFILM Wako Pure Chemical Corporation, Osaka, Japan) using the In-Fusion HD Cloning Kit (Takara Bio, Inc., Shiga, Japan). This plasmid was named pCAG-Neo/RIEDL-wPDPN. DNA encoding the CD20 gene (IRAL012D02) was provided by the RIKEN BRC through the National BioResource Project of MEXT, Japan. The open reading frame of CD20 plus the inserted RIEDL tag between Pro169 and Ala170 of human CD20 was subcloned into a pCAG-Ble vector using In-Fusion HD Cloning Kit. This plasmid was named pCAG-Ble/CD20-_169_RIEDL_170_.

### 2.2 Cell lines

Chinese hamster ovary (CHO)-K1 cells were obtained from the American Type Culture Collection (ATCC, Manassas, VA). CHO-K1 cells were transfected with pCAG-Neo/RIEDL-wPDPN using Lipofectamine LTX with Plus Reagent (Thermo Fisher Scientific Inc., Waltham, MA, USA), and stable transfectants were selected by limiting dilution. The CHO-K1 cells were also transfected with pCAG-Ble/CD20-_169_RIEDL_170_ using Neon Transfection System (Thermo Fisher Scientific, Inc.), and stable transfectants were sorted using a cell sorter (SH800; Sony Corp., Tokyo, Japan). The CHO-K1 cells and transfectants were cultured in RPMI 1640 medium (Nacalai Tesque, Inc., Kyoto, Japan), supplemented with 10% heat-inactivated fetal bovine serum (FBS; Thermo Fisher Scientific Inc.), 100 units/mL of penicillin, 100 μg/mL streptomycin, and 0.25 μg/mL amphotericin B (Nacalai Tesque, Inc.) at 37 °C in a humidified atmosphere containing 5% CO_2_. CHO/RIEDL-wPDPN was cultivated in a medium containing 0.5 mg/ml of G418 (Nacalai Tesque, Inc.). CHO/CD20-_169_RIEDL_170_ was cultivated in a medium containing 0.5 mg/mL zeocin (InvivoGen, San Diego, CA).

### 2.3 Flow cytometry

Cells were harvested by brief exposure to 0.25% trypsin/1 mM ethylenediaminetetraacetic acid (EDTA; Nacalai Tesque, Inc.). After washing with 0.1% bovine serum albumin in phosphate-buffered saline (PBS), cells were treated with primary mAbs (1 μg/ml) for 30 min at 4 °C and subsequently with Alexa Fluor 488-conjugated anti-mouse IgG (1:1000; Cell Signaling Technology, Inc., Danvers, MA, USA). Fluorescence data were collected using an EC800 Cell Analyzer (Sony Corp.).

### 2.4 Expression and purification of RIEDL-tagged proteins

LpMab-7-Sepharose column (2 mg IgG/ml gel) was prepared using CNBr-activated Sepharose 4 Fast Flow (GE Healthcare, Bio-Sciences, Pittsburgh, PA) according to the protocol provided by the manufacturer. The peptide used in the elution was synthesized and purified by reverse-phase HPLC (Sigma-Aldrich Corp., St. Louis, MO). CHO/RIEDL-wPDPN and CHO/CD20-_169_RIEDL_170_ were collected by centrifugation at 190×*g* for 5 min, and lysed with PBS containing 1% Triton X-100 and 0.05 mg/ml aprotinin for 15 min on ice. Supernatant was collected by centrifugation at 22,140×*g* for 15 min, applied to the LpMab-7-Sepharose column (1 ml bed volume), and washed with PBS containing 1% of Triton X-100, followed by washing with PBS containing 0.05% of Tween-20 (PBST). The bound protein was eluted with 0.1 mg/ml decapeptide (RIEDLRIEDL sequence, named 2 × RIEDL peptide), followed by eluting with glycine-HCl buffer (pH 2.7) (Polysciences, Inc., Warrington, PA). In another experiment, the bound protein was eluted with glycine-HCl buffer (pH 2.7), followed by 0.1 mg/ml of 2 × RIEDL peptide.

### 2.5 SDS-PAGE and gel staining

Elution fractions and cell lysates were boiled in sodium dodecyl sulfate (SDS) sample buffer (Nacalai Tesque, Inc.). The samples were electrophoresed on 5%–20% polyacrylamide gels under reducing condition (Nacalai Tesque, Inc.). The gels were stained using Coomassie Brilliant Blue (CBB, Bio-Rad Laboratories, Inc., Berkeley, CA) for 30 min. The gels were also stained using Oriole Fluorescent Gel Stain (Bio-Rad Laboratories, Inc.) for 2 h and were visualized with a Sayaca-Imager (DRC Co., Ltd., Tokyo, Japan).

### 2.6 Western blotting

Elution fractions and cell lysates were boiled in SDS sample buffer (Nacalai Tesque, Inc.). The samples were electrophoresed on 5%–20% polyacrylamide gels (Nacalai Tesque, Inc.), and were transferred onto polyvinylidene difluoride (PVDF) membranes (Merck KGaA, Darmstadt, Germany). After blocking with 4% skim milk (Nacalai Tesque, Inc.) for 1 h, the membranes were incubated with LpMab-7 (1 μg/ml) or PMab-237 (1 μg/ml) for 1 h, followed by HRP-conjugated anti-mouse immunoglobulins (1:1000 dilution; Agilent Technologies, Inc., Santa Clara, CA, USA) for 30 min. The membrane was also incubated with anti-CD20 mAb (EP459Y; 1:1000 dilution; Abcam, Cambridge, UK) for 1 h, followed by anti-rabbit immunoglobulins (1:1000 dilution; Agilent Technologies, Inc.) for 30 min. The membranes were visualized with the ImmunoStar LD Chemiluminescence Reagent (FUJIFILM Wako Pure Chemical Corporation) using the Sayaca-Imager. All procedures of Western blotting were performed at room temperature.

## 3 Results

### 3.1 Establishment of RIEDL tag system for protein purification

We first selected a mAb, LpMab-7, the epitope of which was previously identified as the pentapeptide Arg79, Ile80, Asp81, Glu82, and Leu83 (RIEDL) [16] from our original mAbs library [17]. We named this mAb-peptide tag combination the “RIEDL tag system.” In our previous study, LpMab-7 detected the RIEDL sequence in Western blot, immunohistochemistry, and flow cytometry analyses [16]. In this study, we performed the purification of RIEDL-tagged protein using an LpMab-7-Sepharose column to investigate whether the RIEDL tag system can be applied to protein purification.

**Fig. 1:**
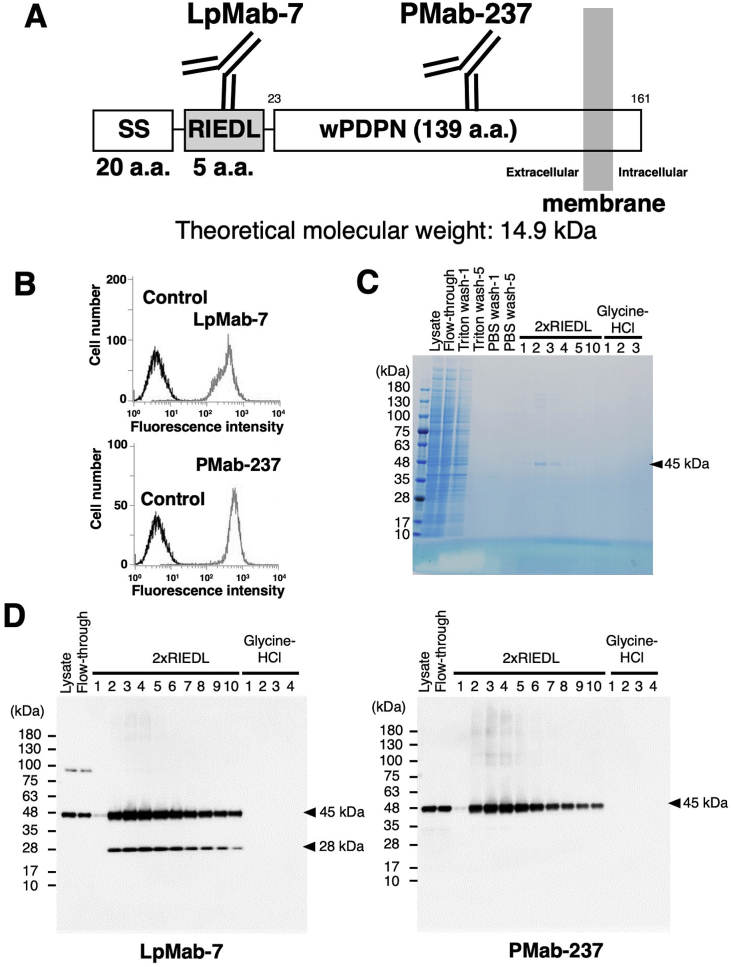
Purification of RIEDL-wPDPN using the RIEDL tag system. (A) Schematic illustration of RIEDL-wPDPN. The epitopes of LpMab-7 or PMab-237 are indicated. SS; signal sequence. (B) Flow cytometry using an anti-RIEDL tag mAb. CHO/RIEDL-wPDPN was detected by an anti-RIEDL tag mAb (LpMab-7, upper panel) and an anti-wPDPN mAb (PMab-237, lower panel). (C) Purification of RIEDL-wPDPN using the RIEDL tag system. Cell lysates (Lysate) were loaded onto an LpMab-7-Sepharose column, and unbound proteins passed through (flow-through). After five washes with PBS containing Triton X-100 (Triton wash-1 and Triton wash-5) and five washes with PBS (PBS wash-1 and PBS wash-5), bound proteins were eluted with 2 × RIEDL peptide (2 × RIEDL 1–5, and -10), followed by acidic buffer (Glycine-HCl 1–3). Elution fractions were applied to SDS-PAGE, and the gel was stained with CBB. (D) Elution fractions from the column chromatography were applied to SDS-PAGE, and the proteins were transferred to PVDF membranes. The membranes were immunostained with 1 μg/ml of an anti-RIEDL tag mAb (LpMab-7; left panel) or an anti-wPDPN mAb (PMab-237; right panel) and incubated with peroxidase-conjugated secondary antibody specific for mouse immunoglobulins.

The RIEDL sequence was added to the N-terminus of wPDPN, and RIEDL-wPDPN was expressed in CHO-K1 cells (Fig. 1A). CHO/RIEDL-wPDPN was detected by anti-RIEDL tag mAb (LpMab-7) and anti-wPDPN mAb (PMab-237) in flow cytometry (Fig. 1B). The lysate of CHO/RIEDL-wPDPN was applied to an LpMab-7-Sepharose column. After five washes with PBS containing Triton X-100, and an additional five washes with PBS, RIEDL-tagged protein was eluted competitively with 2 × RIEDL peptide. The total cell lysate, flow-through from the first column application, several wash fractions (Triton wash-1, Triton wash-5, PBS wash-1, and PBS wash-5), elution fractions using 2 × RIEDL (fraction-1, 2, 3, 4, 5, and 10), and elution fractions using glycine-HCl (fraction-1, 2, and 3) were applied to SDS-PAGE, followed by staining with CBB (Fig. 1C). The 45 kDa RIEDL-wPDPN protein was clearly stained in the 2 × RIEDL peptide elutions, fraction-2 and -3, and weakly detected in the 2 × RIEDL peptide elutions, fraction-1 and -4. In contrast, no protein was observed in the additional glycine-HCl elution fractions (Fig. 1C), indicating that RIEDL-wPDPN was completely eluted by 2 × RIEDL peptide from the LpMab-7-Sepharose column.

Next, we assessed RIEDL-wPDPN protein by Western blot analysis using LpMab-7 (anti-RIEDL) and PMab-237 (anti-wPDPN mAb). As shown in Fig. 1D, LpMab-7 (anti-RIEDL) detected a 45 kDa band (glycosylated) and a 28 kDa band (non-glycosylated) in the 2 × RIEDL elution fractions. In contrast, PMab-237 (anti-wPDPN mAb) detected only the 45 kDa band in the 2 × RIEDL peptide elution fractions. No protein was observed in acidic buffer elution fractions, also indicating that RIEDL-wPDPN was completely eluted by 2 × RIEDL peptide from the LpMab-7-Sepharose column.

### 3.2 Comparison of elution efficiency between 2x RIEDL peptide and acidic buffer

RIEDL-wPDPN was efficiently eluted from the LpMab-7-Sepharose column with 2 × RIEDL peptide, but not eluted by additional glycine-HCl buffer (Fig. 1C), indicating that RIEDL-wPDPN was completely eluted by 2 × RIEDL peptide from the LpMab-7-Sepharose column. To compare the elution efficacy, we changed the elution sequence, where we first eluted using glycine-HCl buffer, followed by a 2 × RIEDL peptide elution. As detected by LpMab-7 in Western blot, RIEDL-wPDPN was clearly eluted using glycine-HCl, but further eluted by an additional 2 × RIEDL peptide elution (Suppl. Fig. 1A). These results were confirmed by Western blot using PMab-237 (Suppl. Fig. 1B). Because the first 2 × RIEDL peptide completely eluted RIEDL-wPDPN from the LpMab-7-Sepharose column, and additional glycine-HCl buffer elution was not necessary (Fig. 1C), an elution procedure using only the 2 × RIEDL peptide was determined to be more efficient and more useful than a glycine-HCl buffer elution.

### 3.3 Insertion of the RIEDL tag into a loop region of CD20

**Fig. 2:**
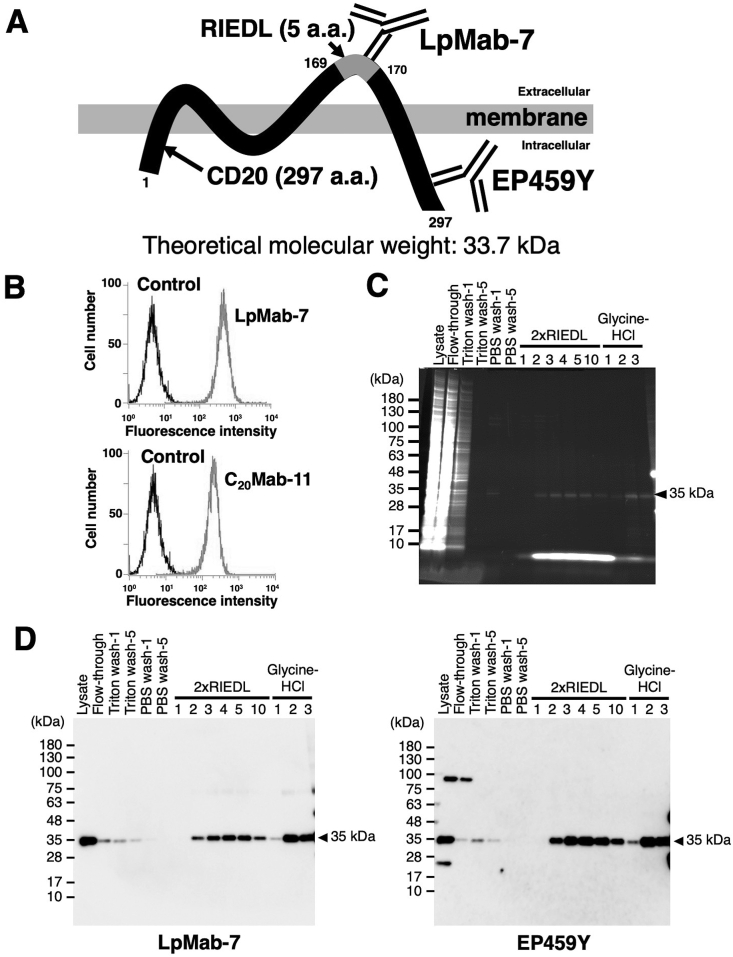
Purification of CD20-_169_RIEDL_170_ using the RIEDL tag system. (A) Schematic illustration of CD20-_169_RIEDL_170_. The epitopes of LpMab-7 and anti-CD20 mAb (clone EP459Y) are indicated. (B) Flow cytometry using an anti-RIEDL tag mAb. CHO/CD20-_169_RIEDL_170_ was detected by anti-RIEDL tag mAb (LpMab-7, upper panel) and anti-CD20 mAb (C_20_Mab-11, lower panel). (C) Cell lysates (Lysate) were loaded onto an LpMab-7-Sepharose column, and unbound proteins passed through (flow-through). After five washes with PBS containing Triton X-100 (Triton wash-1 and Triton wash-5) and five washes with PBS (PBS wash-1 and PBS wash-5), bound proteins were eluted with 2 × RIEDL peptide (2 × RIEDL 1–5, and -10) followed by acidic buffer (glycine-HCl 1–3). Elution fractions were applied to SDS-PAGE, and the gel was stained with Oriole Fluorescent Gel Stain. (D) Elution fractions from the column chromatography were applied to SDS-PAGE, and the proteins were transferred to PVDF membranes. The membranes were immunostained with 1 μg/ml of an anti-RIEDL tag mAb (LpMab-7; left panel) or an anti-CD20 mAb (clone EP459Y; right panel) and incubated with peroxidase-conjugated secondary antibody specific for mouse immunoglobulins.

We investigated whether anti-RIEDL tag mAb (LpMab-7) could still detect the RIEDL tag when the tag was inserted into a loop region of CD20 (Fig. 2A). We inserted the RIEDL sequence between Pro169 and Ala170, and called this construct CD20-_169_RIEDL_170_. In flow cytometry, LpMab-7 detected CD20-_169_RIEDL_170_, which was overexpressed in CHO-K1 cells (Fig. 2B). As a positive control, an anti-CD20 mAb (C_20_Mab-11) also detected CD20-_169_RIEDL_170_.

CD20-_169_RIEDL_170_ was purified using the same purification strategy with RIEDL-wPDPN. Cell lysate, flow-through of the first application, several wash fractions (Triton wash-1, Triton wash-5, PBS wash-1, and PBS wash-5), elution fractions using 2 × RIEDL (fraction-1, 2, 3, 4, 5, and 10), and elution fractions using glycine-HCl (fraction-1, 2, and 3) were applied to SDS-PAGE, followed by staining with Oriole Fluorescent Gel Stain. The 35 kDa protein was detected in the 2 × RIEDL peptide elution fractions (fraction-2, 3, 4, 5, and 10) and in the glycine-HCl elution fractions (fraction-1, 2, and 3), indicating that CD20-_169_RIEDL_170_ was not completely eluted by 2 × RIEDL peptide from the LpMab-7-Sepharose column, and instead could be further eluted using an additional glycine-HCl elution. (Fig. 2C).

Next, we assessed CD20-_169_RIEDL_170_ protein by Western blot analysis using LpMab-7 (anti-RIEDL) and clone EP459Y (anti-CD20 mAb). As shown in Fig. 2D (left panel), LpMab-7 clearly detected a 35 kDa-band in the 2 × RIEDL peptide elution fractions (fraction-2, 3, 4, 5, and 10), and in the glycine-HCl elution fractions (fraction-2 and 3) at a higher intensity, indicating that the glycine-HCl elution is more effective than the 2 × RIEDL peptide elution. Anti-CD20 mAb also detected the same 35 kDa-band (Fig. 2D, right panel).

These results demonstrated that the RIEDL tag, which was inserted into a loop structure of CD20, was sensitively detected by LpMab-7 in flow cytometry, and was useful for the purification of CD20 using LpMab-7.

## 4 Discussion

Tag systems are an essential tool for biological experiments. In this study, we sought to construct a novel tag system by searching for a high affinity mAb with a corresponding small-sized epitope from our original mAbs library [17]. We selected LpMab-7, the epitope of which was identified as the pentapeptide Arg79, Ile80, Asp81, Glu82, and Leu83 (RIEDL) [16]. We named the combination of RIEDL tag and LpMab-7 mAb the “RIEDL tag system.” To investigate the utility of this tag system in protein purification, we purified two RIEDL-tagged proteins, including RIEDL-wPDPN and CD20-_169_RIEDL_170_, using affinity column chromatography with LpMab-7-Sepharose. The RIEDL-tagged proteins were purified by competitive elution with 2 × RIEDL peptide, and the resulting purified proteins exhibited high purity (Figs. 1C and 2C), indicating that RIEDL-tagged proteins can be eluted without denaturation and purified in one step by affinity chromatography.

RIEDL-wPDPN was efficiently and completely eluted using 2 × RIEDL peptide elution from the LpMab-7-Sepharose column, as we observed no further elution with acidic buffer (Fig. 1D). Furthermore, RIEDL-wPDPN could be eluted using 2 × RIEDL peptide even after an acidic buffer elution step (Suppl. Fig. 1), demonstrating that the 2 × RIEDL peptide could elute RIEDL-wPDPN more efficiently than acidic buffer. In contrast, CD20-_169_RIEDL_170_ could not be completely eluted from the LpMab-7-Sepharose column using this method, and required an additional elution step using acidic buffer (Fig. 2D). This result indicates that LpMab-7 might bind more strongly to the RIEDL tag, which was inserted into a loop structure in CD20. Although the 2 × RIEDL peptide did not completely elute CD20-_169_RIEDL_170_ in one step, the RIEDL tag system was shown to be useful not only for detecting CD20 in flow cytometry, but also for purifying CD20 when the RIEDL sequence was inserted into a loop structure of CD20.

The lengths of well-known tags, including FLAG [8], Myc [10], and PA [1], are 8 amino acids, 10 amino acids, and 12 amino acids, respectively. Small peptide tags do not interfere with the structure and function of target proteins; therefore, these tag systems are very useful for detecting functional proteins. However, it was shown that FLAG and Myc tags inserted into loop structures in integrin could not be detected by anti-tag mAbs [1], and as a result, tags are usually added to the N- or C-termini of target proteins. In contrast, the PA tag inserted into loop structure could be detected by an anti-PA mAb, even though this tag is a dodecapeptide [1]. The most critical characteristic of our new RIEDL tag system is its use of a very short peptide tag sequence, the pentapeptide “RIEDL.” Therefore, the RIEDL tag system might be useful not only for detection and purification of transmembrane proteins, but also for further functional biological analyses without interfering with protein function. If needed, we further insert protease recognition sequences between RIEDL tag and proteins. We did not confirm whether the structure or function of RIEDL tagged proteins were affected by inserting RIEDL tag. In the future study, we should perform additional experiments for investigating the structure or function of RIEDL-tagged proteins compared to RIEDL tag-removed proteins.

## Funding

This research was supported in part by 10.13039/100009619AMED [grant numbers: JP20am0401013, JP20am0101078, JP20ae0101028] (Y·K.).

## Declaration of competing interest

The authors declare no conflicts of interest involving this article.

## Figures and Tables

**figs1:**