# Supplementary material for: RIEDL tag: A novel pentapeptide tagging system for transmembrane protein purification
Source: Biochem Biophys Rep. 2020 Jul 17;23:100780. doi: 10.1016/j.bbrep.2020.100780 (PMC7369347; doi:10.1016/j.bbrep.2020.100780)
Supplement: Multimedia component 1 [file mmc1.docx]

**SUPPLEMENTARY INFORMATION**

**Supplementary Figure 1**. Reversal of elution order for RIEDL-wPDPN from a LpMab-7-sepharose column with acidic buffer and 2×RIEDL peptide

Cell lysate (Lysate) was loaded onto a LpMab-7-sepharose column, and unbound proteins passed through (flow-through). After five washes with PBS containing Triton X-100 and five washes with PBS, bound proteins were eluted with acidic buffer (glycine-HCl 1-5, and -10), followed by 2×RIEDL peptide (2×RIEDL 1-8). Elution fractions from the column chromatography were applied to SDS-PAGE, and the proteins were transferred to PVDF membranes. The membranes were immunostained with 1 μg/ml of LpMab-7 (A) or PMab-237 (B) and incubated with peroxidase-conjugated secondary antibody specific for mouse immunoglobulins.
